# Metastasis to Sartorius Muscle from a Muscle Invasive Bladder Cancer

**DOI:** 10.1155/2014/524757

**Published:** 2014-12-23

**Authors:** Ioannis Katafigiotis, Antonios Athanasiou, Panagiotis K. Levis, Evangelos Fragkiadis, Stavros Sfoungaristos, Achilles Ploumidis, Adamantios Michalinos, Christos Alamanis, Evangelos Felekouras, Constantinos A. Constantinides

**Affiliations:** ^1^A University Urology Clinic, University of Athens, Laiko Hospital, 17 Agiou Thoma Street, 11527 Athens, Greece; ^2^Department of Urology, Athens University Medical School, Laiko Hospital, 17 Agiou Thoma Street, 11527 Athens, Greece; ^3^First Department of Surgery, University of Athens Medical School, Athens, Greece; ^4^Department of Urology, Patras University Medical School, Patras, Greece; ^5^Department of Urology, Medical Center, Athens, Greece

## Abstract

Bladder cancer constitutes the ninth most common cancer worldwide and approximately only 30% of cases are muscle invasive at initial diagnosis. Regional lymph nodes, bones, lung, and liver are the most common metastases from bladder cancer and generally from genitourinary malignancies. Muscles constitute a rare site of metastases from distant primary lesions even though they represent 50% of total body mass and receive a large blood flow. Skeletal muscles from urothelial carcinoma are very rare and up to date only few cases have been reported in the literature. We present a rare case of 51-year-old patient with metastases to sartorius muscle 8 months after the radical cystectomy performed for a muscle invasive bladder cancer.

## 1. Introduction

Bladder cancer constitutes the ninth most common cancer worldwide and approximately only 30% of cases are muscle invasive at initial diagnosis [1, 2]. We present a rare case of 51-year-old patient with metastases to sartorius muscle 8 months after the radical cystectomy performed for a muscle invasive bladder cancer.

## 2. Case Report

A 51-year-old male presented with gross macroscopic hematuria, and the ultrasound imaging suggested the presence of a bladder wall tumor, which was confirmed with a cystoscopy. Patient underwent a transurethral resection of the bladder tumor. The pathology report confirmed a pT2 muscle invasive bladder cancer. The computed tomography of the abdomen and pelvis was negative for metastases. After 10 days the patient was subjected to a radical cystoprostatectomy and urinary diversion to an orthotopic ileal neobladder with a modified S-pouch. The final pathology report described a tumor to the posterior wall and the dome of the bladder. The prostate, the seminal vesicles, the distal ureters, and the lymph nodes were free of cancer involvement. The surgical margins were also negative. Patient did not receive chemotherapy in a neoadjuvant or adjuvant setting. The patient 8 months postoperatively complained of left thigh pain and a palpable mass. Thereafter, a full body MRI was performed and revealed a lesion of about 8 cm in diameter in the upper portion of the sartorius muscle, with no other abnormalities or metastatic sites.

**Figure 1:**
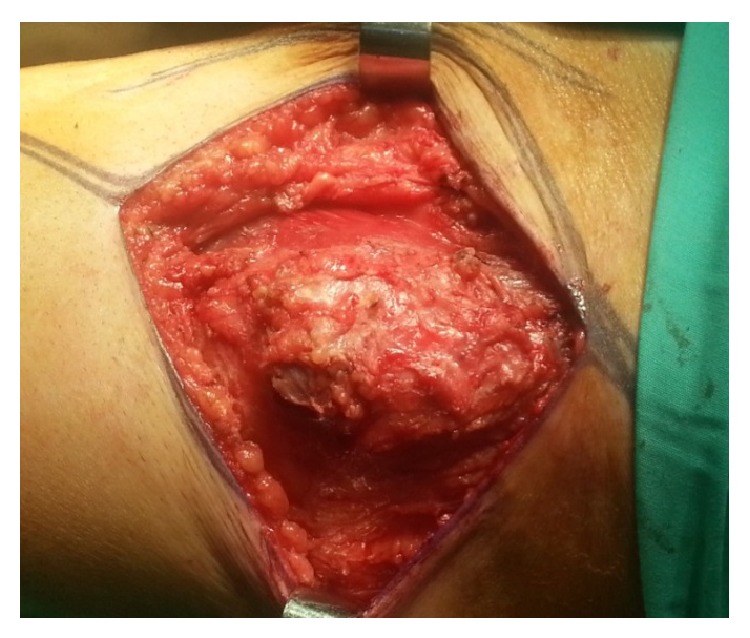
Macroscopic appearance of the metastases from bladder cancer to left sartorius muscle.

**Figure 2:**
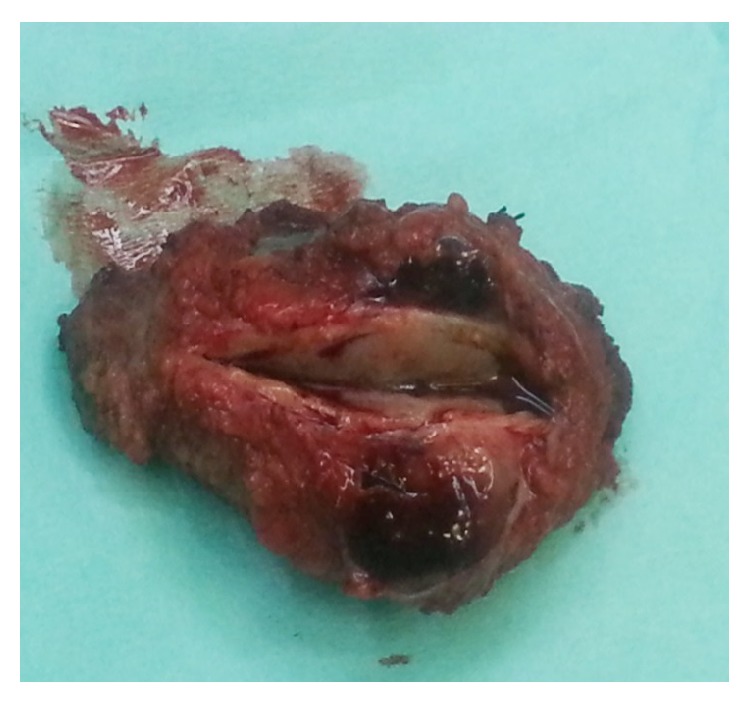
Macroscopic appearance of the metastases from bladder cancer to left sartorius muscle.

**Figure 3:**
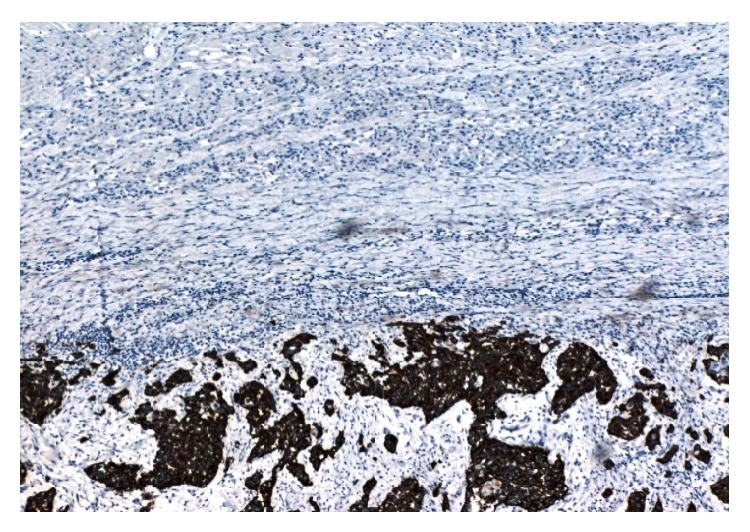
Infiltration of a skeletal muscle from a malignant lesion compatible with a high grade urothelial carcinoma positive to the CK7 marker.

The patient underwent surgical resection of the lesion after induction of general anesthesia. The mass involved only the muscular plane of the sartorius, 10 cm approximately from the origin without evident contact with the tendon or adjacent muscle. A marginal excision of the lesion was performed. Macroscopically, it was an 8 × 4 × 4 cm lesion with smooth margins and the cross-section was white and shiny (Figures 1 and 2). The postoperative period was uneventful. The pathology of the lesion reported a 9 × 4 × 3,9 cm skeletal muscle with a solid lesion of a maximal diameter of 7,2 cm. The skeletal muscle was infiltrated from a malignant lesion compatible with a high grade urothelial carcinoma positive to the CK7 marker and negative to the CK20 (Figure 3). The marker of cellular proliferation Ki67 was 40% positive. The patient subsequently received systematic chemotherapy with gemcitabine and cisplatin (6 cycles) and was subjected to radiotherapy at the site of the excision of the skeletal muscle metastasis and that of the cystectomy. The patient 7 months after the excision of the muscle metastasis in the left thigh is still alive and with a good performance status with a negative imaging follow-up.

## 3. Discussion

Regional lymph nodes, bones, lung, and liver are the most common metastases from bladder cancer and generally from genitourinary malignancies [3]. Less frequent metastatic sites from urothelial carcinoma include the pleura, brain, and skin [4]. Muscles constitute a rare site of metastases from distant primary lesions even though they represent 50% of total body mass and receive a large blood flow [4, 5]. Different factors have been proposed to act protectively against muscle metastases such as muscle pH, muscle contractility, and local changes in oxygenation and lactic acid accumulation that possibly act against tumor neovascularity [6, 7]. On the other hand studies occurring from autopsies show rates of skeletal muscle metastases fluctuating from 6% to 17,5% [6, 8, 9]. This controversial data suggests that skeletal muscles metastases may be more frequent but they occur at an advanced stage of a malignancy and the majority constitute microscopic metastases undetectable from the imaging modalities [4, 5]. Various primary malignant sites have been reported to give muscular metastases such as neoplasms from pancreas, lung, kidney, stomach, colon, and ovaries [4, 5]. Skeletal muscles from urothelial carcinoma are very rare and up to date only few cases have been reported in the literature [4, 5, 10, 11]. Usually the confirmation of the metastases from urothelial carcinoma to a skeletal muscle is achieved via an ultrasound-guided biopsy, but, in our case, both the fact that the mass was painful and in an anatomical position easily susceptible to surgical excision we chose to remove the lesion for diagnosis and treatment purposes [4, 5]. Usually the largest muscles of the body such as the psoas, gluteals, and the erector spinae are the most common sites of the metastases [5]. It is interesting to mention that in one of the cases reported in the literature the left sartorius was the site of the metastases just like in our case [5]. Considering the use of immunohistochemistry in the differential diagnosis of urothelial carcinoma there is no ideal marker or established panel to confirm urothelial differentiation [12]. Positive immunohistochemical markers GATA3, CK20, p63, and either high-molecular weight cytokeratin (HMWCK) or cytokeratin (CK)5/6 can be of value in the differential diagnosis of urothelial carcinoma, while GATA3, S-100P, CK7, CK20, HMWCK, and p63 can help in the diagnosis of the urothelial lineage of variant morphologies [12, 13]. Also considering the contribution of computed tomography in the diagnosis of urothelial carcinoma metastases in a skeletal muscle a low-density, ring-enhancing lesion could be attributed to a metastases until proven otherwise, meaning that during the follow-up of a patient with a bladder cancer a lesion like this should not be overlooked [11]. The suggested method of treating skeletal metastases is chemotherapy with or without the addition of local radiotherapy [5, 11]. Of course this is a palliative way of dealing with pain due to the infiltration of the skeletal muscles from the carcinoma [5, 11]. Although chemotherapy and radiotherapy are usually the preferred palliative treatment method, en bloc excision of the lesion in presence of painful muscle metastases can offer good results [5, 11]. We also performed an open excision of the lesion resulting in a significant improvement of the quality of life of the patient without compromising the use of chemotherapy and radiotherapy. The prognosis of patients with skeletal metastases is poor and the patients survival has a mean duration of 8 months [11]. Our patient 7 months after the surgical excision of the skeletal metastases is still alive with a good performance status.

## References

[1] Ploeg M., Aben K. K. H., Kiemeney L. A. (2009). The present and future burden of urinary bladder cancer in the world. *World Journal of Urology*.

[2] Vaidya A., Soloway M. S., Hawke C., Tiguert R., Civantos F. (2001). De novo muscle invasive bladder cancer: Is there a change in trend?. *Journal of Urology*.

[3] Babaian R. J., Johnson D. E., Llamas L., Ayala A. G. (1980). Metastases from transitional cell carcinoma of urinary bladder. *Urology*.

[4] Nagao E., Nishie A., Yoshimitsu K., Irie H., Shioyama Y., Naito S., Matsuura S., Honda H. (2004). Gluteal muscular and sciatic nerve metastases in advanced urinary bladder carcinoma: case report. *Abdominal Imaging*.

[5] Doo S. W., Kim W. B., Kim B. K., Yang W. J., Yoon J. H., Song Y. S., Choi I. H. (2012). Skeletal muscle metastases from urothelial cell carcinoma. *Korean Journal of Urology*.

[6] Acinas García O., Fernández F. A., Satué E. G., Buelta L., Val-Bernal J. F. (1984). Metastasis of malignant neoplasms to skeletal muscle. *Revista Espanola de Oncologia*.

[7] Mulsow F. W. (1943). Metastatic carcinoma of skeletal muscles. *Archives of Pathology*.

[8] Pickren J. W., Weiss L. (1976). Use and limitations of autopsy data. *Fundamental Aspects of Metastasis*.

[9] Pearson C. M. (1959). Incidence and type of pathologic alterations observed in muscle in a routine autopsy survey. *Neurology*.

[10] Ekici S., Özen H., Gedikoglu G., Aygün C. (1999). Skeletal muscle metastasis from carcinoma of the bladder. *Scandinavian Journal of Urology and Nephrology*.

[11] Nabi G., Gupta N. P., Gandhi D. (2003). Skeletal muscle metastasis from transitional cell carcinoma of the urinary bladder: clinicoradiological features. *Clinical Radiology*.

[12] Amin M. B., Trpkov K., Lopez-Beltran A., Grignon D., Members of the ISUP Immunohistochemistry in Diagnostic Urologic Pathology Group (2014). Best practices recommendations in the application of immunohistochemistry in the bladder lesions: report from the International Society of Urologic Pathology consensus conference. *The American Journal of Surgical Pathology*.

[13] Paner G. P., Annaiah C., Gulmann C., Rao P., Ro J. Y., Hansel D. E., Shen S. S., Lopez-Beltran A., Aron M., Luthringer D. J., De Peralta-Venturina M., Cho Y., Amin M. B. (2014). Immunohistochemical evaluation of novel and traditional markers associated with urothelial differentiation in a spectrum of variants of urothelial carcinoma of the urinary bladder. *Human Pathology*.

